# First-in-Human Study of [^211^At]NaAt as Targeted α-Therapy in Patients with Radioiodine-Refractory Thyroid Cancer (Alpha-T1 Trial)

**DOI:** 10.2967/jnumed.125.270810

**Published:** 2025-12

**Authors:** Tadashi Watabe, Kosuke Mukai, Sadahiro Naka, Hidetaka Sasaki, Takashi Kamiya, Tomoaki Hayakawa, Atsunori Fukuhara, Toru Takano, Yoshifumi Shirakami, Kazuhiro Ooe, Satoshi Shigeno, Satomi Okamura, Kazuho Masumura, Eisuke Hida, Hiromitsu Haba, Atsushi Toyoshima, Kayako Isohashi, Iichiro Shimomura, Noriyuki Tomiyama

**Affiliations:** 1 Department of Radiology, Graduate School of Medicine, University of Osaka, Osaka, Japan;; 2 Institute for Radiation Sciences, University of Osaka, Osaka, Japan;; 3 Department of Metabolic Medicine, Graduate School of Medicine, University of Osaka, Osaka, Japan;; 4 Department of Pharmacy, University of Osaka Hospital, Osaka, Japan;; 5 Department of Medical Technology, University of Osaka Hospital, Osaka, Japan;; 6 Department of Gastroenterology and Hepatology, Graduate School of Medicine, University of Osaka, Osaka, Japan;; 7 Medical Innovation Center, University of Osaka Hospital, Osaka, Japan;; 8 Department of Biostatistics and Data Science, Graduate School of Medicine, University of Osaka, Osaka, Japan; and; 9 RIKEN Nishina Center for Accelerator-Based Science, Wako, Japan

**Keywords:** astatine, [^211^At]NaAt, targeted α-therapy, radioactive iodine, ^131^I, differentiated thyroid cancer

## Abstract

^211^At, a cyclotron-produced α-emitter, has attracted interest as a potential alternative to radioactive iodine (RAI) because of its superior cytotoxic properties. This first-in-human prospective clinical trial aimed to evaluate the safety and preliminary efficacy of [^211^At]NaAt in patients with differentiated thyroid cancer (DTC). **Methods:** Eleven patients with metastatic RAI-refractory DTC were enrolled in this study. A single intravenous dose of [^211^At]NaAt was administered in the setting of recombinant human thyroid-stimulating hormone stimulation and an iodine-restricted diet. Dose escalation followed a modified 3 + 3 design, with doses of 1.25 (*n* = 2), 2.5 (*n* = 3), and 3.5 MBq/kg (*n* = 6). The primary endpoint was the assessment of adverse events using Common Terminology Criteria for Adverse Events version 5.0 guidelines and dose-limiting toxicities. Secondary endpoints included evaluation of pharmacokinetics, absorbed dose, and therapeutic efficacy. **Results:** Dose-limiting toxicities occurred in 3 of 6 patients who received 3.5 MBq/kg, consisting of grade 3 hematologic toxicity (lymphopenia or leukopenia) lasting for more than 1 wk. Other major adverse events included salivary gland swelling (predominantly grade 2), xerostomia (grades 1 and 2), nausea (grade 2), decreased appetite (grade 2), and vomiting (grade 2). A reduction of greater than 50% in recombinant human thyroid-stimulating hormone–stimulated thyroglobulin levels was observed in 1 of 3 patients (33%) who received 2.5 MBq/kg and 2 of 5 patients (40%) who received 3.5 MBq/kg. At 6 mo, CT-based evaluation showed stable disease (SD) in 9 patients (90%) and progressive disease in 1 patient (10%). ^131^I SPECT imaging revealed SD in the only patient who received 1.25 MBq/kg. Of the 3 patients treated with 2.5 MBq/kg, a partial response was seen in 1 patient (33%) and SD in 2 patients (67%). Of the 5 patients who received 3.5 MBq/kg, complete response, partial response, and progressive disease were noted in 1 patient (20%) each, and SD was found in 2 patients (40%). **Conclusion:** Targeted α-therapy with [^211^At]NaAt was well tolerated and showed preliminary efficacy in patients with RAI-refractory DTC, supporting further clinical investigations.

## Visual Abstract

**Figure:**
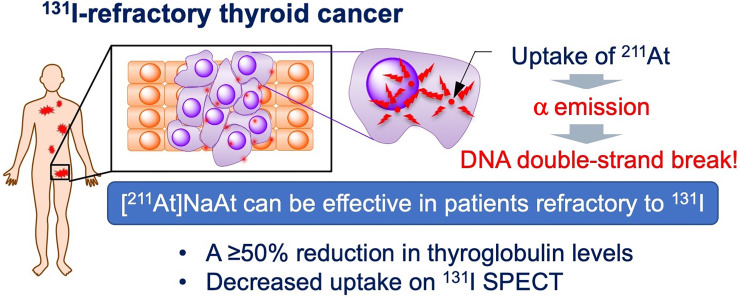

In differentiated thyroid cancer (DTC), including papillary and follicular carcinomas, the sodium iodide symporter is expressed similar to normal thyroid tissue, allowing for the uptake of radioactive iodine (RAI) (1). Currently, RAI therapy using [^131^I]NaI is the standard treatment for patients with recurrent or metastatic DTC after total thyroidectomy (2). However, despite repeated administration, some patients do not achieve sufficient therapeutic effects and experience disease progression (3). According to the American Thyroid Association guidelines, patients with DTC are classified as refractory to RAI when there is disease progression after RAI treatment, even in the presence of RAI uptake, which is also encountered in clinical practice (4). Although molecular-targeted agents are the standard treatment for RAI-refractory DTC, they require daily oral administration and are associated with a high rate of adverse effects, including proteinuria and dermatologic toxicity (5). Therefore, there is a clinical need for next-generation radiopharmaceutical therapy with improved tolerability and efficacy that can be used to treat RAI-refractory patients.

^211^At is a halogen element with properties similar to iodine and exhibits comparable biodistribution, including uptake by the thyroid and thyroid cancer via the sodium iodide symporter (6). ^211^At is an α-particle emitter with a physical half-life of 7.2 h and can be produced using a 30-MeV cyclotron (7). α-particles release high energy over a short range, resulting in a higher linear energy transfer than conventional β-particles. In preclinical studies using thyroid cancer cell lines, ^211^At demonstrated superior induction of DNA double-strand breaks compared with ^131^I, along with enhanced inhibition of colony formation, significant tumor regression, and prolonged survival in tumor-bearing mouse models (6,8).

In prostate-specific membrane antigen–targeted radiopharmaceutical therapy for prostate cancer, α-particle therapy with ^225^Ac has demonstrated high efficacy in patients refractory to β-particle therapy with ^177^Lu (9,10). This suggests that α-particle therapy with astatine, an iodine analog, may also be effective in patients who are refractory to conventional RAI therapy using β-particle emitters. In many countries, including Japan, high-dose RAI therapy with [^131^I]NaI requires inpatient isolation in specialized radiation treatment rooms (11). However, a shortage of such rooms has led to prolonged waiting times before treatment initiation, potentially resulting in further disease progression (12). These challenges underscore the need for therapeutic options that can be used in outpatient settings.

We aimed to develop a targeted α-therapy using [^211^At]NaAt to address the unmet clinical needs of RAI-refractory DTC. In this first-in-human investigator-initiated phase 1 trial, we evaluated the adverse events (AEs), pharmacokinetics, absorbed dose, and therapeutic efficacy of a single intravenous dose of [^211^At]NaAt in patients with RAI-refractory DTC.

## MATERIALS AND METHODS

This first-in-human phase 1 study (NCT05275946) was conducted at the University of Osaka Hospital in compliance with the Good Clinical Practice Guidelines of the International Conference on Harmonization. The protocol was approved by the institutional review board (approval 219004), and all patients gave written informed consent.

### Patient Selection

Patients were included if they had RAI-refractory DTC (papillary or follicular subtype) that showed an insufficient therapeutic response after more than 3 courses of [^131^I]NaI treatment, regardless of prior administration of molecular targeted agents, or if they were unable or unwilling to undergo a second or subsequent RAI treatment or standard therapies. Other eligibility criteria included age greater than 18 y; an Eastern Cooperative Oncology Group performance status of 0–2; a stable general condition with a life expectancy of more than 6 mo; and the following laboratory values: white blood cell count of at least 3,000/mm³, absolute neutrophil count of 1,500/mm³ or greater, platelet count of at least 100,000/mm³, hemoglobin of 9.0 g/dL or greater, total bilirubin not exceeding 2.4 mg/dL, aspartate and alanine transaminase of no more than 120 units/L, serum creatinine not exceeding 1.5 mg/dL, and creatinine clearance of 50 mL/min or greater. Patients were excluded if they had multiple active cancers or received chemotherapy, immunotherapy, or radiation therapy within 8 wk before study enrollment.

### Study Design

This phase 1 dose-escalation study followed a modified 3 + 3 design, with doses of 1.25, 2.5, 3.5, 5, 7, and 10 MBq/kg. These doses were determined in accordance with the International Council for Harmonisation S9 guideline and on the basis of a preclinical toxicity study of [^211^At]NaAt (13). A dose-limiting toxicity (DLT) was defined as any toxicity occurring within 4 wk after the administration of [^211^At]NaAt that met 1 or more of the following criteria: grade 3 hematologic toxicity persisting for at least 7 d; grade 4 or higher hematologic toxicity, regardless of duration; febrile neutropenia, regardless of duration; thrombocytopenia accompanied by bleeding tendency or requiring platelet transfusion; anemia requiring the transfusion of red blood cells; neutropenia accompanied by infection; and grade 3 or higher nonhematologic toxicity persisting for 7 d or longer and not alleviated by symptomatic treatment. All toxicities were graded in accordance with the Common Terminology Criteria for Adverse Events version 5.0.

### Preparation of [^211^At]NaAt and Treatment Protocol

The [^211^At]NaAt solution was prepared in compliance with Good Manufacturing Practice standards, as previously reported (14). ^211^At was separated and purified via dry distillation from bismuth plates irradiated via the ^209^Bi(^4^He, 2n)^211^At nuclear reaction using an azimuthally varying field cyclotron at the RIKEN Nishina Center for Accelerator-Based Science. After purification, ^211^At was collected in a solution containing 1% ascorbic acid and 2.3% sodium hydrogen carbonate. The radiochemical purity of the resulting [^211^At]At^−^ was 99% ± 1%. γ-ray spectrometry using a high-purity germanium detector confirmed the presence of ^211^At-specific peaks and the absence of ^210^At contamination, thereby verifying the radionuclidic purity of ^211^At.

Before administration of [^211^At]NaAt, a pretreatment protocol similar to that used for RAI therapy was applied (4). Iodine restriction was implemented from 2 wk before until 2 days after the administration of [^211^At]NaAt. Recombinant human thyroid-stimulating hormone (rhTSH; Thyrogen; Sanofi), was administered intramuscularly at a dose of 0.9 mg into the gluteal muscle at 48 and 24 h before the administration of [^211^At]NaAt (15). A single dose of [^211^At]NaAt was administered by slow intravenous injection (mean volume, 7.3 mL) over approximately 1 min, followed by a 20-mL saline flush, after which patients could leave the radioisotope-controlled area. Isolated hospitalization in a dedicated room was not required (16).

The primary endpoints were the evaluation of AEs and DLTs as measures of safety and tolerability. The secondary endpoints included vital signs, body temperature, body weight, subjective and objective clinical symptoms, blood and urine tests, electrocardiograms, changes in blood concentrations, pharmacokinetics, and assessment of excretion. The clinical trial period continued for up to 6 mo after [^211^At]NaAt administration. The use of other anticancer therapies, including molecular-targeted agents, was prohibited during the study period.

Whole-body SPECT/CT scans were performed at 1, 3, and 24 h after the administration of [^211^At]NaAt using a VERITON-CT system (Spectrum Dynamics Medical), targeting characteristic x-rays (79 keV) emitted from the daughter nuclide of ^211^Po (17). Regions of interest were manually delineated in major organs using PMOD software (PMOD Technologies Ltd.), and residence times were derived from time–activity curves using the trapezoidal integration method. Subsequently, the absorbed doses were calculated using the internal dosimetry software (IDAC-Dose 2.1) (18).

### Response Evaluation

Tumor response was preliminarily and exploratorily evaluated using serum thyroglobulin, CT, and [^131^I]NaI SPECT/CT.

#### Thyroglobulin Measurement

Thyroglobulin with rhTSH stimulation was used as a tumor marker. Changes in thyroglobulin levels at 3 and 6 mo after the administration of [^211^At]NaAt were compared with the baseline levels obtained during screening. If rhTSH stimulation was not feasible, the unstimulated thyroglobulin values were used.

#### CT Imaging

Diagnostic CT scans were performed at screening and 3 and 6 mo after administration of [^211^At]NaAt. Changes in the size of the metastatic lesions on CT were assessed according to RECIST version 1.1.

#### ^131^I SPECT Imaging

Diagnostic [^131^I]NaI scans (74 MBq) were performed with rhTSH stimulation at the time of screening and 3 and 6 mo after the administration of [^211^At]NaAt using the Symbia Intevo 6 system (Siemens Healthineers). Changes in uptake were assessed visually based as a complete response (CR; no significant abnormal uptake indicative of metastasis or recurrence), partial response (PR; marked visual decrease in abnormal uptake compared with the baseline scan), progressive disease (PD; marked visual increase in abnormal uptake compared with the baseline scan or the appearance of new lesions), or stable disease (SD; cases that do not meet the criteria for CR, PR, or PD and show no significant visual change in uptake). As semiquantitative references, target-to-normal ratio and SUV were measured. Up to 2 target regions per organ (maximum of 5 lesions per patient) were evaluated. PR was defined as a decrease of 50% or more in total target-to-normal ratio or SUV, whereas PD was characterized by an increase of 50% or more in total target-to-normal ratio or SUV.

### Statistical Analysis

Statistical analyses were performed using datasets from the Study Data Tabulation Model and Analysis Dataset Model, prepared in accordance with Clinical Data Interchange Standards Consortium standards, using SAS software (version 9.4; SAS Institute Inc.). Pharmacokinetic parameter estimation was performed using WinNonlin software (version 8.4; Certara).

## RESULTS

Eleven patients were enrolled in this study between February 2022 and May 2024. Patient characteristics are summarized in Table 1. [^211^At]NaAt dose escalation resulted in the administration of 1.25 MBq/kg in 2 patients, 2.5 MBq/kg in 3 patients, and 3.5 MBq/kg in 6 patients. DLTs were not observed patients receiving 1.25 or 2.5 MBq/kg but were experienced by 3 of 6 patients who received 3.5 MBq/kg. The observed DLTs were hematologic toxicities, including grade 3 lymphopenia (*n* = 2) and leukopenia (*n* = 1), which persisted for more than 1 wk. Treatment-related AEs are summarized in Table 2. Hematologic toxicities included anemia (grade 1 in 6 patients), leukopenia (grade 2 in 3 patients and grade 3 in 4 patients), lymphopenia (grade 2 in 2 patients and grade 3 in 7 patients), neutropenia (grade 2 in 3 patients and grade 3 in 2 patients), and thrombocytopenia (grade 1 in 2 patients). Other major AEs included salivary gland swelling (grade 1 in 1 patient and grade 2 in 7 patients), xerostomia (grade 1 in 4 patients and grade 2 in 2 patients), nausea (grade 2 in 8 patients), decreased appetite (grade 2 in 3 patients), vomiting (grade 2 in 4 patients), and diarrhea (grade 1 in 3 patients). No clinically significant changes were observed in patients’ vital signs, urinalysis results, or electrocardiograms. No severe AEs were reported.

**TABLE 1.** Characteristics of Study Patients (*n* = 11)

Characteristic	Value
Sex	
Female	7
Male	4
Age (y)	60 (34–79)
Body weight (kg)	56 (47–89)
ECOG performance status	0
Histologic subtype	
Papillary	3 (27%)
Follicular	8 (73%)
Time from initial diagnosis (y)	6.0 (3.9–18.0)
Total thyroidectomy	11 (100%)
Site of metastasis	
Lung	8 (73%)
Bone	7 (64%)
TSH suppression therapy	10 (91%)
No. prior RAI treatments*	
1^†^	1 (9%)
3 or more	10 (91%)
Prior multikinase inhibitor therapy	
Lenvatinib	1 (9%)
Prior radiation therapy	4 (36%)
Prior immune-checkpoint inhibitor	
Pembrolizumab	1 (9%)

* Mean total dose of ^131^I was 15.3 ± 3.9 GBq.

† Total dose was 3.7 GBq

Continuous data are expressed as median, followed by range in parentheses.

ECOG = Eastern Cooperative Oncology Group; TSH = thyroid-stimulating hormone.

**TABLE 2.** Treatment-Related AEs, by [^211^At]NaAt Dose, According to Common Terminology Criteria for Adverse Events Version 5.0

	1.25 MBq/kg (*n* = 2)		2.5 MBq/kg (*n* = 3)		3.5 MBq/kg (*n* = 6)		Total (*n* = 11)	
AE	Grade 1 or 2	Grade 3 or 4	Grade 1 or 2	Grade 3 or 4	Grade 1 or 2	Grade 3 or 4	Grade 1 or 2	Grade 3 or 4
Anemia	0	0	2	0	4	0	6 (55)	0
Leukopenia	1	0	0	0	2	4*	3 (27)	4 (36)
Lymphopenia	0	0	2	1*	0	6*	2 (18)	7 (64)
Neutropenia	1	0	0	0	2	2*	3 (27)	2 (18)
Thrombocytopenia	1	0	1	0	0	0	2 (18)	0
Salivary gland swelling	0	0	2	0	6	0	8 (73)	0
Xerostomia	0	0	2	0	4	0	6 (54)	0
Nausea	1	0	2	0	5	0	8 (73)	0
Decreased appetite	0	0	0	0	3	0	3 (27)	0
Vomiting	0	0	2	0	2	0	4 (36)	0
Diarrhea	0	0	0	0	3	0	3 (27)	0
Taste disorder	0	0	0	0	1	0	1 (9)	0
Fatigue	0	0	0	0	1	0	1 (9)	0
Increased serum bilirubin	0	0	0	0	1	0	1 (9)	0
Headache	0	0	0	0	1	0	1 (9)	0
Neck pain	1	0	0	0	0	0	1 (9)	0

* Grade 3.

Data expressed as number or number and percentage.

**FIGURE 1.**
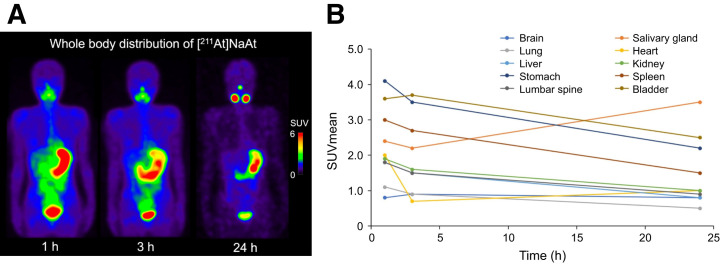
(A) Representative SPECT maximum-intensity-projection images in patient with follicular thyroid cancer after administration of [^211^At]NaAt (3.5 MBq/kg). Physiologic accumulation was observed in salivary glands, stomach, and bladder, with rapid clearance from blood and background tissues. At 24 h after administration, increased uptake was noted in submandibular gland, accompanied by swelling. No significant uptake was detected in metastatic lesions in this patient. (B) Time course of [^211^At]NaAt distribution in major organs.

Representative whole-body SPECT/CT images after the administration of [^211^At]NaAt and time–activity curves are shown in Figures 1A and 1B, respectively. At 3 h after administration, physiologic uptake (SUV_mean_) was observed in the stomach (3.47 ± 1.35), bladder (3.62 ± 0.68), spleen (2.70 ± 0.51), and salivary glands (2.21 ± 0.66). Urine is the primary route of excretion. Although imaging suggested that ^211^At was also excreted into the gastric juice and subsequently entered the digestive tract, fecal excretion remained minimal within 24 h after administration. In addition, excretion via expired air was negligible. Blood concentrations, pharmacokinetic parameters, and excretion data are summarized in Supplemental Tables 1–7, available at http://jnm.snmjournals.org. The dosimetric results are presented in Table 3. Relatively high absorbed doses were observed in the stomach (2.75 ± 1.46 mGy/MBq) and salivary glands (1.96 ± 0.80 mGy/MBq), whereas red bone marrow had an absorbed dose of 0.57 ± 0.15 mGy/MBq.

**TABLE 3.** Mean Absorbed Dose in Major Organs After Administration of [^211^At]NaAt

Organ	Absorbed dose (mGy/MBq)
Brain	0.17 ± 0.04
Salivary gland	1.96 ± 0.81
Lung	0.002 ± 0.000
Heart	0.14 ± 0.03
Liver	0.18 ± 0.03
Kidney	0.33 ± 0.08
Stomach	2.75 ± 1.46
Spleen	0.38 ± 0.17
Bone marrow	0.57 ± 0.15
Bladder	0.003 ± 0.001

Of the 2 patients who received 1.25 MBq/kg of [^211^At]NaAt, 1 patient discontinued participation in the clinical trial 3 mo after administration because of the need for an alternative treatment; therefore, efficacy data were available only from the CT scan performed at 3 mo after administration. Additionally, postadministration ^131^I SPECT was not performed for 1 of the 6 patients who received 3.5 MBq/kg, and thyroglobulin levels were evaluated without rhTSH stimulation.

**FIGURE 2.**
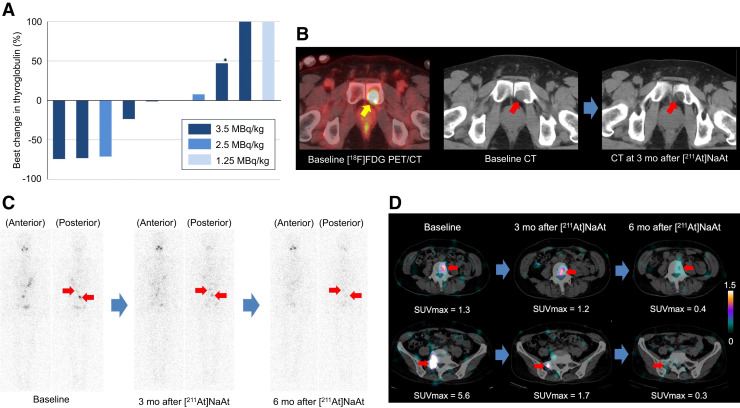
(A) Waterfall plot showing maximum change in serum thyroglobulin levels at 3 or 6 mo after administration of [^211^At]NaAt. Asterisk indicates level measured without rhTSH stimulation. (B) Representative images of patient with follicular thyroid cancer with metastasis in left pubic bone before and after administration of [^211^At]NaAt (2.5 MBq/kg). Tumor shrinkage of [^18^F]FDG-avid lesion was observed on CT. (C and D) Representative ^131^I planar and SPECT/CT images in patient with follicular thyroid cancer with multiple bone metastases at baseline and 3 and 6 mo after administration of [^211^At]NaAt (3.5 MBq/kg). ^131^I accumulation in lumbar spine and sacral metastases gradually decreased over time.

A waterfall plot of the best response of thyroglobulin levels after administration of [^211^At]NaAt is presented in Figure 2A. A reduction in thyroglobulin of more than 50% was not observed in patients treated with 1.25 MBq/kg but was observed in 1 of 3 patients (33%) who received 2.5 MBq/kg and 2 of 6 patients (33%) treated with 3.5 MBq/kg. Response evaluation results using CT and ^131^I SPECT are summarized in Table 4. CT-based response evaluations conducted 6 mo after administration showed SD in the 1 patient treated with 1.25 MBq/kg and SD in all 3 patients who received 2.5 MBq/kg. Of the 6 patients treated with 3.5 MBq/kg, SD was found in 5 patients and PD in 1 patient. ^131^I SPECT imaging at 6 mo revealed SD in the 1 patient treated with 1.25 MBq/kg and PR in 1 of 3 and SD in 2 of 3 patients who received 2.5 MBq/kg. Among the 6 patients treated with 3.5 MBq/kg, 5 had evaluable ^131^I SPECT images. Of these, 1 each demonstrated CR, PR, and PD, whereas 2 were found to have SD. Figures 2B–2D show representative cases of tumor shrinkage on CT and decreased ^131^I uptake in bone metastases on SPECT. Comprehensive data on patient background, dosage, and treatment response are presented in Supplementary Tables 8–12.

**TABLE 4.** Response Evaluation Using CT and ^131^I SPECT Data at 6 Months After Administration of [^211^At]NaAt

Patient	Dose	CT	^131^I SPECT	Comment
1	1.25 MBq/kg	SD	SD	No significant change in ^131^I uptake in skull bone metastasis
2	1.25 MBq/kg	NA*	NA*	
3	2.5 MBq/kg	SD	PR	73% decrease in SUV_max_ of left rib metastasis
4	2.5 MBq/kg	SD	SD	No significant change in ^131^I uptake in bone metastases
5	2.5 MBq/kg	SD	SD	No significant change in mild uptake in some lung metastases
6	3.5 MBq/kg	SD	PR	90% decrease in total SUV_max_ of bone metastases
7	3.5 MBq/kg	SD^†^	NA^‡^	No apparent ^131^I uptake in lung metastases
8	3.5 MBq/kg	SD^†^	SD	No significant change in ^131^I uptake in multiple tiny lung metastases
9	3.5 MBq/kg	PD	SD	Progression of multiple lung metastases without ^131^I uptake; no significant change in bone metastases
10	3.5 MBq/kg	SD^†^	PD	New uptake in left occipital bone; increased uptake in left lung metastases
11	3.5 MBq/kg	SD^†^	CR	Disappearance of uptake in skull bone metastasis

* Evaluation not performed because participation was terminated 3 mo after administration.

† Classified as non-CR/non-PD because of the absence of target lesions in accordance with RECIST version 1.1.

‡ Evaluation not performed because of the absence of uptake on ^131^I SPECT.

NA = not available.

## DISCUSSION

In this first-in-human study, we demonstrated that targeted α-therapy using [^211^At]NaAt can be safely administered to patients with DTC. Although DLTs were observed with the 3.5 MBq/kg dose, toxicities remained within a tolerable range. Preliminary evidence of efficacy was observed in some patients treated with either 2.5 or 3.5 MBq/kg, including thyroglobulin reductions of greater than 50% and decreased uptake in RAI-avid lesions on ^131^I SPECT.

During dose escalation, the DLTs consisted of leukopenia and lymphopenia lasting more than 1 wk. Since bone marrow and lymphocytes are highly radiosensitive, a decline in lymphocyte count was observed as early as the day after administration, likely because of the cytotoxic effects of α-particles emitted by astatine (19). However, in most cases, leukocyte and lymphocyte counts returned to baseline over time. Regarding leukopenia, an average decrease of 43% in white blood cells from baseline was observed 1 wk after administration. However, a clear recovery trend was evident, with reductions improving to 22% at 2 wk and 7% at 4 wk, with complete recovery observed from 8 wk onward. Because these changes were transient and asymptomatic, they were considered tolerable.

Physiologic accumulation of [^211^At]NaAt was notable in SPECT imaging, and relatively high absorbed doses were observed in the stomach (2.75 ± 1.46 mGy/MBq) and salivary glands (1.96 ± 0.81 mGy/MBq). At doses of 2.5 MBq/kg or higher, treatment-related AEs included nausea, vomiting, decreased appetite, salivary gland swelling, and xerostomia. Among these symptoms, nausea, vomiting, and decreased appetite typically resolved within 2–3 d, although they persisted for more than 1 wk in some cases. Salivary gland swelling was transient and resolved within a few days, whereas xerostomia was common and associated with a sustained reduction in saliva secretion. Xerostomia has also been reported in prostate-specific membrane antigen–targeted α-therapies attributable to physiologic accumulation in the salivary glands, suggesting their high susceptibility to α-radiation (20,21). Cooling of the salivary glands with ice packs was performed in this study before dose administration until 2–3 d after administration. Although the effectiveness of salivary gland cooling in reducing xerostomia remains unclear, it appears to have some effect in alleviating swelling.

We used rhTSH stimulation as a pretreatment to enhance drug accumulation by elevating thyroid-stimulating hormone levels, consistent with standard RAI therapy. rhTSH has been reported to be as effective as thyroid hormone withdrawal for thyroid-stimulating hormone stimulation (22). Moreover, thyroid hormone withdrawal may make it difficult to distinguish between hypothyroidism symptoms and AEs related to [^211^At]NaAt administration (23). Therefore, we selected the rhTSH protocol for this study to enable a more accurate evaluation of AEs. However, thyroid hormone withdrawal remains an established approach in patients with metastatic disease, and future clinical trials should further investigate the optimal strategy for thyroid-stimulating hormone stimulation (4). In addition, whereas thyroglobulin levels were measured under rhTSH stimulation in this study, thyroid-stimulating hormone–suppressed thyroglobulin may be sufficient for future studies.

In this trial, DLTs was observed in 3 of 6 patients who received 3.5 MBq/kg, indicating that the maximum tolerated dose was likely exceeded, according to conventional criteria (24). In addition, considering AEs related to physiologic accumulation in the salivary glands and gastrointestinal tract—such as xerostomia, nausea, vomiting, and decreased appetite—the recommended dose for future phase 2 trials is proposed to be 2.5 MBq/kg as a single administration dose. However, since many current radiopharmaceutical therapies require repeated dosing (3–6 cycles), repeated administration should be explored in future trials to enhance therapeutic efficacy (20).

Regarding the efficacy evaluation, most patients in this study had multiple bone or lung metastases. Bone metastases are difficult to evaluate using RECIST; in some cases, lung metastases are smaller than 10 mm in diameter, making them nonmeasurable according to RECIST. For cases in which only nontarget lesions were identified, the response was classified as non-CR/non-PD unless clear progression, the appearance of new lesions, or CR was observed. Even in patients with measurable lung metastases, no PR or greater reduction in lesion size was confirmed, highlighting the need to optimize treatment protocols for [^211^At]NaAt in future trials. Furthermore, progression-free survival and clinical remission with an extended follow-up period should be assessed to determine the clinically meaningful efficacy of treatment in subsequent clinical trials.

This clinical trial included patients with progressive DTC with a history of 3 or more RAI treatments; however, the definitions of RAI refractoriness and PD were not clearly established. Standardized definitions should be clarified in future clinical trials. Iodine avidity was not included in the formal inclusion criteria. Nevertheless, patients with some degree of ^131^I-avidity were selected on the basis of the uptake observed during their previous RAI treatments. In addition, ^131^I SPECT was performed as a screening test to evaluate RAI accumulation.

RAI accumulation, which reflects sodium iodide symporter expression, varies among metastatic lesions, and sodium iodide symporter expression tends to decrease over the course of RAI treatment (4). Therefore, further investigation of sodium iodide symporter redifferentiation therapies using *BRAF* or *MEK* inhibitors may be warranted to enhance the accumulation of [^211^At]NaAt, whereas careful consideration should be given to the potential risk of increased hematotoxicity (25,26). Nevertheless, a reduction in thyroglobulin levels of 50% or greater was observed in 1 of 3 patients who received 2.5 MBq/kg and 2 of 6 treated with 3.5 MBq/kg. Similarly, decreased or absent uptake in RAI-avid lesions was observed on ^131^I SPECT in 33% patients in both of these cohorts. These findings suggest that targeted α-therapy with [^211^At]NaAt can be effective in patients with RAI-refractory DTC.

## CONCLUSION

Targeted α-therapy with [^211^At]NaAt was well tolerated and showed preliminary efficacy in patients with RAI-refractory DTC. Further investigation is warranted to comprehensively evaluate its therapeutic potential.

## DISCLOSURE

This study was funded by the AMED Clinical Research and Trial Promotion Research Project (grant 23lk0201139h0003) and Alpha-Fusion, Inc. Tadashi Watabe received research funding from Alpha-Fusion Inc. No other potential conflict of interest relevant to this article was reported.

## Notes

KEY POINTS **QUESTION:** Can [^211^At]NaAt be used for targeted α-therapy in patients with DTC refractory to conventional RAI therapy? **PERTINENT FINDINGS:** A single intravenous dose of [^211^At]NaAt was well tolerated, with some patients exhibiting a reduction in thyroglobulin levels of 50% or greater and decreased uptake on ^131^I imaging, suggesting its therapeutic potential. **IMPLICATIONS FOR PATIENT CARE:** [^211^At]NaAt is a promising α-emitter for ^131^I-refractory DTC, potentially offering a treatment option before the use of molecular-targeted agents.
